# Management of Spontaneous Renal Arteriovenous Fistula in Pregnancy

**DOI:** 10.31486/toj.24.0062

**Published:** 2025

**Authors:** Comfort S. Tamakloe, Conner Davey, Kaitlyn Dorn, Patrick Gilbert, Frank B. Williams

**Affiliations:** ^1^ The University of Queensland Medical School, Ochsner Clinical School, New Orleans, LA; ^2^ Department of Urology, Ochsner Clinic Foundation, New Orleans, LA; ^3^ Department of Maternal-Fetal Medicine, Ochsner Clinic Foundation, New Orleans, LA; ^4^ Department of Radiology, Ochsner Clinic Foundation, New Orleans, LA

**Keywords:** *Arteriovenous fistula*, *embolization–therapeutic*, *pregnancy complications*

## Abstract

**Background:**

A renal arteriovenous fistula (RAVF) is an abnormal connection between the artery and vein of the kidney and can result from idiopathic, congenital, or traumatic causes. In the general population, this pathology is rare and has the potential to be life-threatening. The incidence in pregnancy is even rarer.

**Case Report:**

A pregnant 33-year-old gravida 5, para 4 patient presented with gross hematuria and passage of clots and was diagnosed with a right renal lower pole arteriovenous fistula at 36 weeks, 6 days’ gestation. The patient developed gestational hypertension but did not demonstrate severe features of preeclampsia She underwent labor induction, uncomplicated spontaneous vaginal delivery, and subsequent endovascular embolization of the fistula. Hematuria and hypertension resolved postembolization.

**Conclusion:**

Because RAVF presents with nonspecific symptoms that mimic other causes of hematuria and hypertension in pregnancy, this rare vascular anomaly poses challenges in diagnosis and management in pregnant patients. Our case was also challenging because of the late-stage pregnancy diagnosis. The case highlights the challenges of diagnosing and managing RAVF during pregnancy and underscores the importance of a multidisciplinary approach.

## INTRODUCTION

Renal arteriovenous fistula (RAVF) is an abnormal connection between a renal artery and vein. Formation of an arteriovenous fistula involves an injury or disruption of the vascular walls that allows blood to bypass the capillary bed and flow directly from the arterial to the venous circulation.^1^ While the majority of RAVF cases are asymptomatic, patients can present with resistant hypertension, gross hematuria, renal insufficiency, and hemorrhage.^2^ We report a case of hematuria and worsening hypertension in a pregnant patient diagnosed with right-sided RAVF.

## CASE REPORT

A 33-year-old gravida 5, para 4 patient with anemia of pregnancy presented to the emergency department (ED) with gross hematuria and passage of clots at 36 weeks and 6 days of gestation. She had been evaluated a week prior for similar symptoms in the ED affiliated with her primary obstetrician's office. At the prior presentation, her vital signs were stable, and hemoglobin was stable at 10.6 g/dL (reference range, 11.0-16.0 g/dL for pregnant females in the third trimester),^3^ up from 8.9 g/dL 3 weeks prior following initiation of iron supplementation. A urologist had been consulted at that time, although further hematuria evaluation was deferred until after pregnancy. Urine culture showed >100,000 CFU/mL *Escherichia coli*. The patient received a 7-day course of cephalexin for possible acute cystitis, but her symptoms did not resolve.

She presented to the authors’ ED for further evaluation and a second opinion, reporting continued hematuria with associated right-sided flank pain and lower pelvic pain. She denied headache, visual disturbances, shortness of breath, and right upper quadrant pain. She was not taking anticoagulants or antiplatelet medication. Her medical history was remarkable for previous nephrolithiasis and mild asthma. She had no history of previous surgical intervention, instrumentation, or trauma to the right kidney. Her pregnancy history was remarkable for previous gestational hypertension and 4 spontaneous vaginal term deliveries.

On initial assessment, blood pressure was elevated at 147/89 mm Hg. Otherwise, the patient's vital signs were stable and unremarkable. Abdominal examination was normal with no appreciable thrills, and her kidneys were not palpable. Reflexes were appropriate without clonus, and lung fields were clear. Anemia was again present, with hemoglobin of 9.4 g/dL, hematocrit of 28% (reference range, 36%-46% for adult females), and platelet count of 244,000/mm^3^ (reference range, 150,000-400,000/mm^3^). Creatinine was normal at 0.6 mg/dL (reference range, 0.6-1.2 mg/dL); urine protein assessment was deferred in light of frank hematuria. Liver enzymes were normal. Fetal nonstress test was reactive and reassuring.

**Figure 1.**
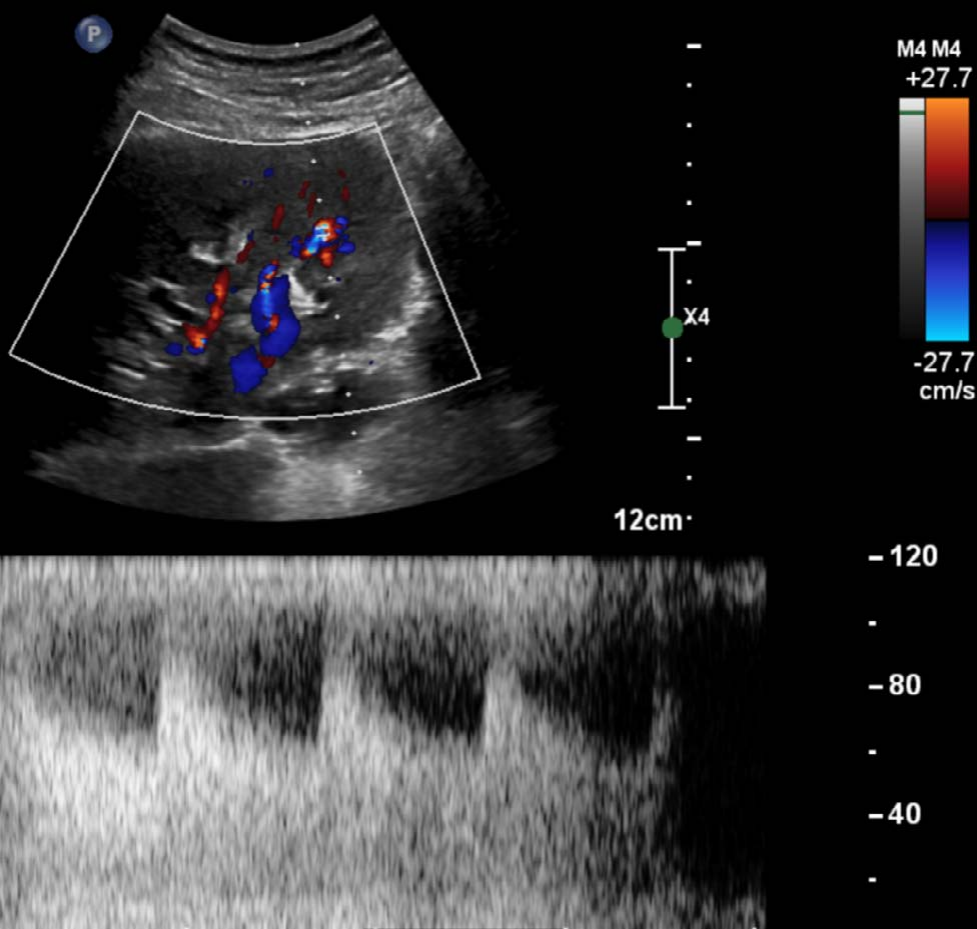
Ultrasound of the right kidney shows turbulent flow in the lower pole with abnormal waveforms, consistent with a renal arteriovenous fistula. Although resistive indices were not measured, they were subjectively low, further corroborating the presence of a renal arteriovenous fistula.

Retroperitoneal Doppler ultrasound showed an unremarkable left kidney. The right kidney measured 12.9 cm, and a 3.1 × 3.0 × 3.6-cm right upper pole complex cystic lesion with septations and nodular wall thickening demonstrated flow to the wall thickening but no concerning hypervascularity. An area of turbulent flow in the right lower pole was consistent with a right-sided RAVF (Figure 1).

The consulting urologist recommended computed tomography angiography (CTA) and endovascular intervention but noted that the procedure could be temporarily deferred given the patient's clinical stability. However, the urologist warned that hemorrhage associated with RAVF was possible. Overnight observation was recommended, with serial assessment of hemoglobin and hematocrit.

Interval blood pressures were intermittently mildly elevated (ranges of 121-149/74-91 mm Hg), establishing a diagnosis of gestational hypertension, but the patient did not demonstrate severe features of preeclampsia. Given the patient's newly diagnosed gestational hypertension and persistent hematuria with anemia in the context of RAVF, the decision was made to induce labor. Vaginal delivery was planned because of the fewer risks and complications for the mother compared to cesarean section.

**Figure 2.**
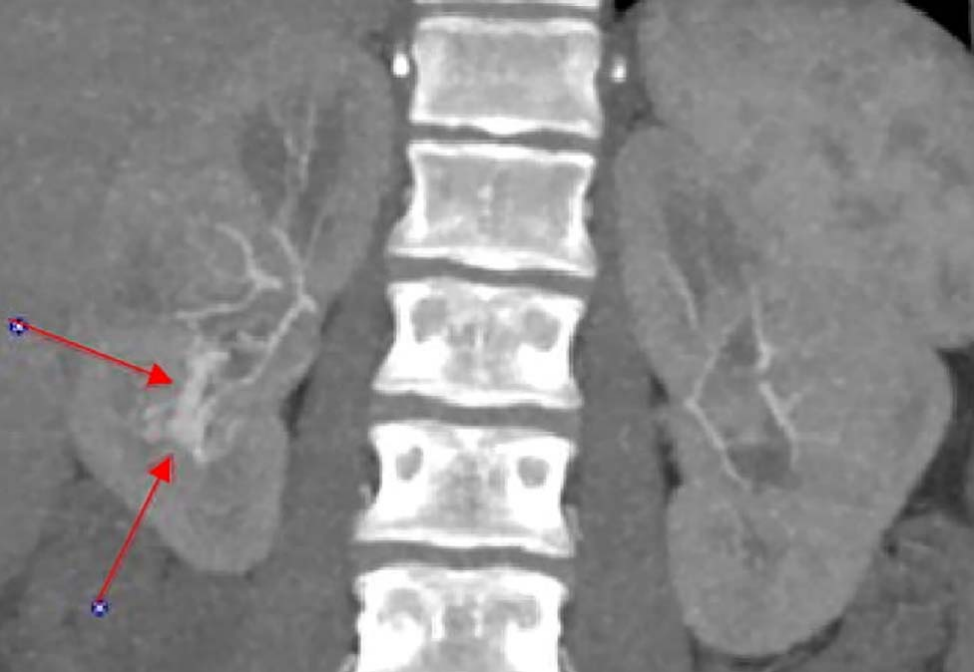
Computed tomography angiography shows early filling of the right renal vein in the lower pole (arrows), corresponding to the renal arteriovenous fistula.

**Figure 3.**
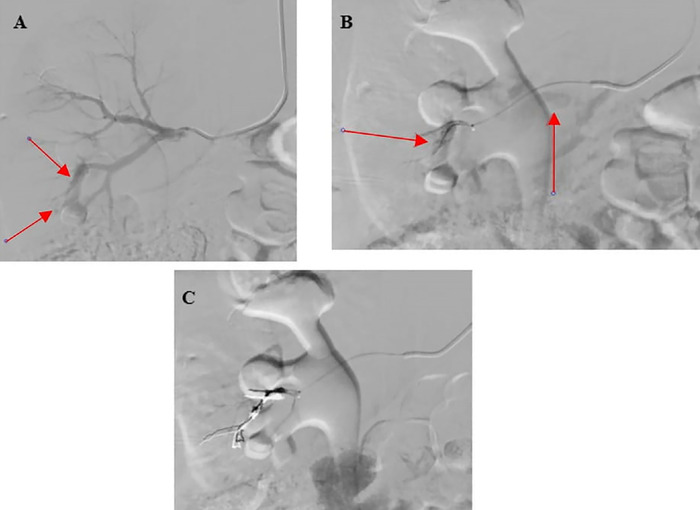
(A) Right renal angiogram shows abnormal vasculature of the lower pole with early opacification of the lower pole renal vein (arrows). (B) Selection of the tertiary branch of the inferior branch of the right renal artery shows similar findings of an abnormal tangle of vessels and filling of the renal vein (arrows). (C) Postprocedure coil embolization of the small feeding artery shows no further identification of the renal arteriovenous fistula.

Following an uncomplicated spontaneous vaginal delivery on hospital day 2, CTA performed on postpartum day 1 demonstrated a small tangle of slightly prominent vessels at the inferior pole of the right kidney with early opacification of the right renal vein compared to the left renal vein, confirming a small arteriovenous fistula (Figure 2 and Figures 3A and 3B). Imaging also showed 3 hypoattenuating lesions.

**Figure 4.**
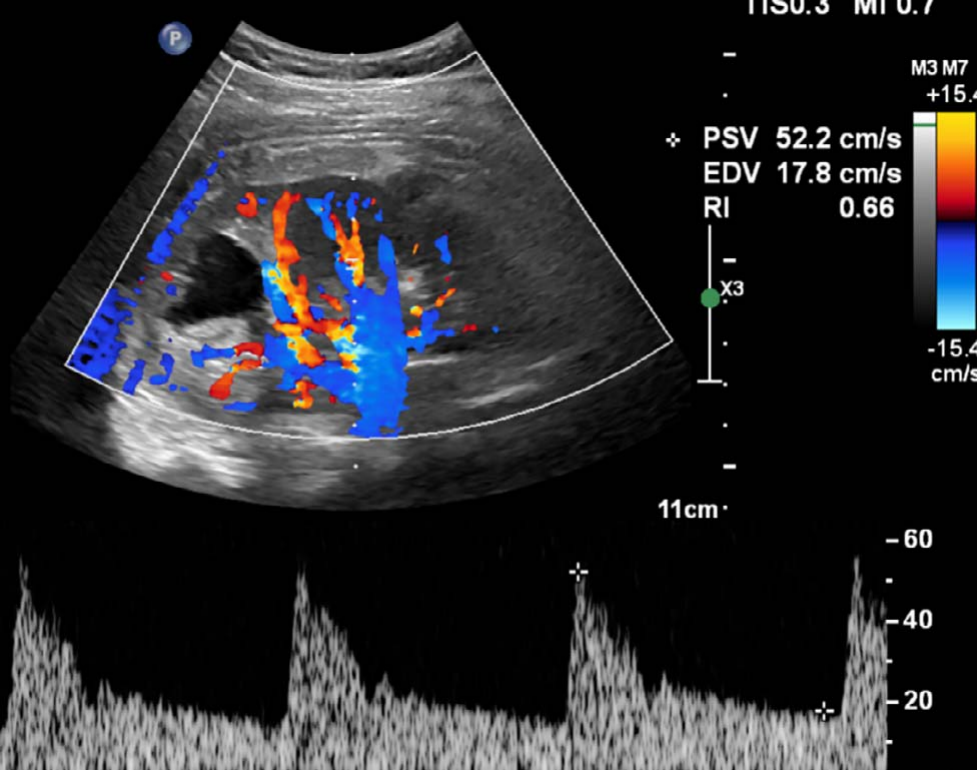
Postintervention ultrasound shows normal waveforms and resistive indices, without evidence of the previously seen turbulent abnormal flow. Echogenic material is noted in the treated renal arteriovenous fistula region, corresponding to the metallic coils used during embolization.

On postpartum day 1, the patient underwent endovascular intervention. Postintervention angiography showed no abnormal arteriovenous communication of the right renal artery (Figure 3C), and postintervention ultrasound revealed the absence of turbulent abnormal flow (Figure 4).

Following the intervention, the patient had a hemoglobin level of 9.6 g/dL and a hematocrit of 27.9%. Hematuria resolved. A postprocedure basic metabolic panel that included creatinine level would have been a useful way to assess the patient's renal function but was not obtained in this case.

During her 4-day hospital stay and at discharge, the patient remained hypertensive. At her 1-week postpartum appointment, the patient had persistently elevated systolic blood pressure readings, ranging from 150 to 159 mm Hg, and she was started on extended-release (XL) nifedipine 30 mg daily. At that time, her hematuria had resolved. At her 3-week postpartum visit, the patient's blood pressure was within the target range (104-145/84-94 mm Hg), and nifedipine XL was continued. At the 6-week postpartum visit, the patient remained asymptomatic, and her blood pressure had fully normalized (106/62 mm Hg), so nifedipine XL was discontinued.

## DISCUSSION

RAVF is linked to the exacerbation of hypertension because of the abnormal vascular connection that disrupts the normal regulation of blood pressure through the renin-angiotensin-aldosterone system.^4^ Because RAVF presents with nonspecific symptoms that mimic other causes of hematuria and hypertension in pregnancy, the diagnosis can be difficult.^5^ The differential diagnosis can include nephrolithiasis, hypertensive disorders of pregnancy, and urinary tract infection.^6,7^

RAVF can be congenital or acquired. Most cases are iatrogenic, occurring as a complication from nephrostomy, renal surgery, or renal biopsy.^2^ Malignancy, inflammation, and blunt/penetrating abdominal trauma have also been implicated in the acquisition of RAVF.^8^

Imaging is extremely important in the diagnosis and subsequent management of RAVF.^8^ While digital subtraction angiography is the gold standard for diagnosis, the procedure is invasive, and other imaging modalities should be considered initially.^8^ When RAVF is suspected, Doppler ultrasound should be first-line imaging, as Doppler ultrasound can show the anomalous communication between the artery and vein and the presence of complications such as thrombosis and aneurysms.^8^ Ultrasonography can differentiate simple cysts from complicated cysts, and color Doppler ultrasound can demonstrate blood flow direction and turbidity of the cystic lesion. If ultrasound raises suspicion for a fistula, assessment via CTA can characterize the presence, location, size, and flow of the fistula.

Treatment approaches depend on the severity of symptoms and potential complications and include close monitoring in asymptomatic patients, minimally invasive endovascular embolization, and surgical repair for large fistulas. Pregnancy-induced hemodynamic changes, such as systemic vasodilation, could cause or exacerbate RAVF by increasing the pressure within blood vessels, potentially leading to complications such as hypertension or preeclampsia, thrombosis, pulmonary embolism, or hemorrhage.^9^ When left untreated, RAVF can progress to high-output cardiac failure, further exacerbating cardiac complications.^10^ To tailor an effective management plan, investigating alternative causes beyond pregnancy-induced hypertension is crucial when a pregnant patient presents with high blood pressure. Indications for treating a fistula include recurrent hematuria, progression of fistula size, hypertension, circulatory overload, and high-output heart failure.^8,11^

Managing RAVF during pregnancy involves carefully considering the treatment of the mother's symptoms while also prioritizing the safety of the fetus. Given the complexity of the condition, a multidisciplinary approach that includes obstetricians, urologists, and interventional radiologists is warranted. The aim of RAVF treatment is to preserve renal function and prevent and treat complications, such as renal failure, cardiac failure, and rupture of dilated veins.^11^ Transcatheter embolization with superselective arterial embolization is a documented effective approach for the minimally invasive treatment of RAVF and is generally considered the treatment of choice for RAVF in the general population.^12,13^ In the context of pregnancy, 2 case reports provide evidence for the safe and effective use of embolization to treat RAVF.^14,15^ Wortman et al reported a successful embolization procedure at 17 weeks’ gestation using low-dose fluoroscopy,^14^ while Yao et al described a case of superselective embolization at 12 weeks’ gestation.^15^

Embolization with low-dose fluoroscopy is favored for treating RAVF in pregnancy because it has fewer complications compared to surgical intervention, effectively preserves renal function, and allows for continuation of pregnancy while minimizing the risks associated with radiation exposure. Although endovascular intervention has been successful in early-term pregnancies, we decided to perform the intervention postpartum to minimize potential harm to the fetus because our patient was near term.

In patients for whom embolization is not successful or the pathology is too complex, surgery, including nephrectomy, direct ligation, and repair of the fistula, might be explored. The case reported by Manogran et al shows the limitations of conservative management, as complications such as progression of the size of the aneurysm/fistula and subsequent erosion into the ovarian vein can require urgent surgical intervention.^16^

Close monitoring postintervention is necessary to assess the effectiveness of treatment and to identify any complications. Renal function should be closely monitored, and blood pressure control should be implemented. Patients should be advised to watch for signs of recurrent RAVF, such as hypertension, hematuria, or flank pain.

## CONCLUSION

In pregnancy, RAVF is a rare yet potentially life-threatening condition that requires swift diagnosis and management. RAVF can present as hypertension, hematuria, or flank pain and can progress to cardiac or renal failure if not managed appropriately. Our case, involving a 33-year-old female diagnosed with RAVF at 36 weeks and 6 days of gestation, highlights the complexities of treatment during pregnancy. Unlike other reported cases in which early intervention was possible, this case was challenging because of the late-stage pregnancy diagnosis. The successful outcome of the case also shows the importance of a multidisciplinary approach when treating RAVF in pregnant patients.

## Notes

*This article meets the Accreditation Council for Graduate Medical Education and the American Board of Medical Specialties Maintenance of Certification competencies for Patient Care and Medical Knowledge.*
